# Sigma: multiple alignment of weakly-conserved non-coding DNA sequence

**DOI:** 10.1186/1471-2105-7-143

**Published:** 2006-03-16

**Authors:** Rahul Siddharthan

**Affiliations:** 1 Institute of Mathematical Sciences, CIT Campus, Taramani, Chennai 600113, India

## Abstract

**Background:**

Existing tools for multiple-sequence alignment focus on aligning protein sequence or protein-coding DNA sequence, and are often based on extensions to Needleman-Wunsch-like pairwise alignment methods. We introduce a new tool, Sigma, with a new algorithm and scoring scheme designed specifically for non-coding DNA sequence. This problem acquires importance with the increasing number of published sequences of closely-related species. In particular, studies of gene regulation seek to take advantage of comparative genomics, and recent algorithms for finding regulatory sites in phylogenetically-related intergenic sequence require alignment as a preprocessing step. Much can also be learned about evolution from intergenic DNA, which tends to evolve faster than coding DNA. Sigma uses a strategy of seeking the best possible gapless local alignments (a strategy earlier used by DiAlign), at each step making the best possible alignment consistent with existing alignments, and scores the significance of the alignment based on the lengths of the aligned fragments and a background model which may be supplied or estimated from an auxiliary file of intergenic DNA.

**Results:**

Comparative tests of sigma with five earlier algorithms on synthetic data generated to mimic real data show excellent performance, with Sigma balancing high "sensitivity" (more bases aligned) with effective filtering of "incorrect" alignments. With real data, while "correctness" can't be directly quantified for the alignment, running the PhyloGibbs motif finder on pre-aligned sequence suggests that Sigma's alignments are superior.

**Conclusion:**

By taking into account the peculiarities of non-coding DNA, Sigma fills a gap in the toolbox of bioinformatics.

## Background

Alignment of homologous biological sequence has long been a central problem in bioinformatics, dating back to the 1970s with the Needleman-Wunsch algorithm for pairwise global alignment [1]. The related Smith-Waterman algorithm [2] dealt with the case of finding pairwise local homology, and these algorithms form the basis of most methods in use today. The general approach for multiple alignment is to build it up up from several pairwise alignments. Many tools, like ClustalW [3] and MLagan [4], align entire sequences globally pairwise (and may require a phylogenetic tree as input to decide in what order to do the pairwise alignments). An alternative approach, pioneered by DiAlign [5,6], is to construct a global multiple alignment from multiple *gapless *local alignments. This requires scoring the significance of local alignments (to decide in what order to make them), and also a consistency check for each local alignment after the first (the assumption being that the sequences being aligned have not been "shuffled" and aligned pieces are syntenous). More sophisticated algorithms, such as T-Coffee [7] and Align-m [8], have since been developed.

All these tools are designed for alignment of proteins or protein-coding DNA. Thus, they use well-established substitution matrices, and perhaps higher-level structural information, when dealing with amino acids, but tend to assume that all nucleotides are alike – or that a substitution matrix (usually derived from coding DNA) adequately describes the differences – when dealing with DNA, or at best use codon translations – inappropriate for non-coding DNA – to search for "anchors". Moreover, they (with the notable exception of DiAlign) tend to penalise insertions and deletions rather severely, which may again be ill-advised for non-coding DNA. Thus, on the one hand, much of the approach of sophisticated protein-alignment algorithms is unnecessary or inappropriate for DNA, while on the other hand, some simpler considerations that apply to non-coding DNA are not used.

A need for tuning an alignment program to non-coding DNA has probably not been felt because it mutates much faster than coding sequence and, often, has diverged too far for any significant homology to survive. Till recently, there were not many sequences of closely-related organisms available. That situation has now changed dramatically. For example, the regulatory regions of five fully-sequenced *sensu stricto *species of yeast *Saccharomyces cerevisiae, S. paradoxus, S. bayanus, S. mikatae, S. kudriavzveii *[9,10] are substantially conserved; many related species of fruitfly, starting with *Drosophila melanogaster *and *D. pseudoobscura*, have been fully or partially sequenced; and mammalian genomes exhibit much homology with one another (and often even with non-mammalian vertebrates) in their non-coding DNA. Accurately aligning orthologous noncoding DNA is now important. PhyloGibbs, a Gibbs sampler that we recently developed for phylogenetically related sequence [11,12], uses multiple alignment of input sequence as a preprocessing step (this was a major motivation for the present work), as do two other such recent programs, PhyMe [13] and EMnEM [14]. (These programs have respectively preferred DiAlign, Lagan and ClustalW as their alignment tools, but these tools are interchangeable apart from some minor, easily-altered details of file format.) Other studies have used "phylogenetic footprinting" (for example, [9,15,16]), that is, assuming that functional sites are concentrated in conserved regions as reported by multiple alignment programs.

Comparative genomics also tells us a lot about evolution, and non-coding DNA is of peculiar importance here precisely *because *it evolves so much faster than genes themselves. It is likely that the major differences in mammals, for example, or in two species of fruitfly or yeast, arise not so much from different genes as from different regulation of essentially the same genes. Moreover, there is probably new and unexpected information buried in the vast quantities of non-coding DNA that mammalian genomes contain. To take into account the peculiarities of this problem, Sigma ("Simple greedy multiple alignment"), the tool presented here, uses a correlated "background model" extracted from actual DNA. Its performance on synthetic data generated from such models is a significant improvement over existing programs. Like DiAlign, it imposes no gap penalty, so sequences that are only partly conserved may still be aligned. Furthermore, its algorithm and scoring are such that the significance of later alignments is increased by the presence of earlier alignments, so that adding more related species actually makes alignment easier. Philosophically it is rather close to DiAlign, but it is a new approach whose algorithm and scoring are unbiased by earlier efforts' focus on proteins.

## Implementation

The core idea of Sigma is the same as that of DiAlign: it builds up a global alignment from significant local (pairwise) gapless alignments and doesn't worry about gaps in the global alignment.

Since sufficiently short local alignments always exist, it is important to score them correctly. Sigma uses an estimate of the probability of seeing such an alignment between random sequences of the same lengths as the given sequences (which is also done, a bit differently, by version 2 of DiAlign). Moreover, Sigma accounts for correlations naturally occurring in DNA sequence, so that one is more likely to align rarely-occurring strings and less likely to align commonly-occurring strings such as poly-A stretches. We demonstrate the practical importance of this in the section on synthetic data.

DiAlign builds a list of possible local alignments ("diagonals") in one pass and then performs a recursively-determined consistent subset of this list in a later pass. In contrast, Sigma always immediately performs the best available local alignment consistent with previous alignments. This improves the sensitivity of Sigma: the significance of later, "smaller" alignments may be increased by the constraints from previously-performed alignments.

**Figure 1:**
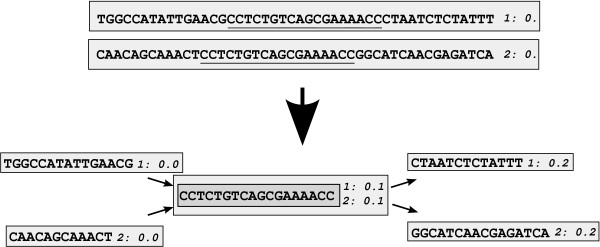
**Alignment of sequence fragments**. Two sequences, initially in their own sequence fragments and labelled "0.", are locally aligned: the aligned piece goes into one fragment with two sequence labels, and the remaining pieces go into their own fragments. The sequence labels increase from left to right on any sequence, and are used to maintain consistency in alignments.

Sigma operates, not on the raw input sequence, but on a set of "sequence fragments" that are labelled in such a way that inconsistent alignments can instantly be rejected. Initially, each input sequence is its own fragment with its own label, and there are no consistency conditions. When a local alignment is performed, the aligned regions of two fragments are merged into one single fragment, while the remaining regions remain as their own fragments (figure 1). At each iteration, the best available local alignments are made, and then the fragment labels and consistency conditions are updated to prevent inconsistent alignments. The iteration terminates when there are no possible local alignments above the specified significance threshold.

### The algorithm: building a global alignment out of local alignments

Each "sequence fragment" contains sequence belonging to one input sequence, or locally-aligned sequence belonging to more than one input sequence. The sequence fragment is a data structure that contains

• A list of the sequences {*S*} that it belongs to. (Initially, each input sequence is an entire sequence fragment and each fragment belongs to exactly one input sequence.)

• A label *L*_*s *_for each sequence *s *in {*S*}, that identifies it on that sequence. (That is, each sequence fragment always has a unique value, for each sequence *s *in {*S*}, of the pair (s, L_*s*_)). The labels are strings representing real numbers between 0 and 0.3, and are in increasing order as one moves along any particular sequence.

• For each sequence *s *in {*S*}, pointers to the previous and next fragments in *s*.

• For every sequence *s' not *in {*S*}, "limits" *L*_*l*_(*s'*) and *L*_*r*_(*s'*) for the left-most and right-most fragments in *s' *with which it may be aligned.

**Figure 2:**
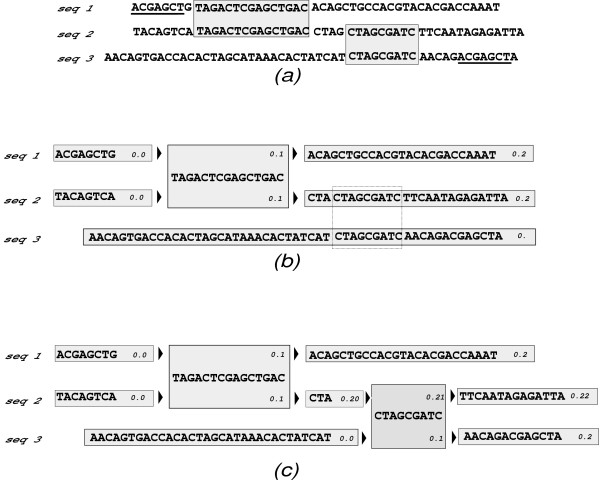
**Enforcing consistency of multiple alignments**. When aligning more than two sequences, care must be taken that new local alignments are compatible with existing alignments. (a) If the two alignments in shaded blocks are already made, the underlined sequence fragments cannot be aligned. (b) The sequence fragments when one local alignment has already been made, and the next (dotted lines) is about to be made. (c) The sequence fragments after the second local alignment has been made. At each stage, each sequence fragment has limits on what other fragments it can be aligned with in every other sequence. For example, in (b), the fragment 0.2 in seq 2 can only be aligned with fragments > 0.1 in seq 1, but the fragment 0. in seq 3 can be aligned with any part of seq 1. After the local alignment is made in (c), the frag 0.1 in seq 3 inherits the more stringent limit from its paired frag in seq 2: it can only be aligned with seq 1 > 0.1, and this limit is moreover "propagated" right to seq 3 frag 0.2. Likewise, the "right-hand" limits (none in this case) are propagated to seq 3 frag 0.0 which means, in this case, that seq 3 frag 0.0 can be aligned to any fragment in seq 1.

The algorithm is:

• Initially there is one fragment for each input sequence, containing the entire sequence, with predecessor and successor elements set to NULL, the label set to 0., and without limits on alignment (any fragment may be aligned with any other).

• Each pairwise alignment operates on two sequence fragments *f*_*i *_and *f*_*j*_, whose sequence sets {*S*}_*i *_and {*S*}_*j *_are disjoint, and which fall within each other's "alignment limits". A possible alignment is an ungapped local alignment between the corresponding sequence stretches.

• A list of all possible pairwise alignments is made, and sorted and performed in order of significance. Alignments inconsistent with prior alignments are omitted.

• When an alignment is performed, the aligned stretch of sequence is broken into its own fragment, resulting in five new fragments (figure 1). These fragments are re-labelled by appending the single digits 0, 1, and 2 to all labels on, respectively, the unaligned pieces on the left, the aligned fragment and the unaligned pieces on the right: see figure 1. Then the allowed alignment limits are updated, which can be done easily in linear time (see below, and figure 2).

• This is done repeatedly until there are no more possible alignments within the specified significance limit.

• The sequence fragment set is then converted into the desired output format and printed.

Thus, the sequence labels and limits enforce the consistency conditions continuously: each fragment has a unique label for each sequence it belongs to, and fragment A can only be aligned to fragment B if (a) they belong to no sequences in common and (b) for each sequence *S *to which fragment B belongs, the label *L*(*S*) is within the limits *L*_*l*_(*S*), *L*_*r*_(*S*) for that sequence on fragment A (and vice versa). The updating of the alignment limits is done by "sweeps" from the newly-aligned fragment *f*_new_. Let {*S*_*f*_} be the set of sequences that *f*_new _is a part of, and {*S'*} the complement of {*S*_*f*_}, that is, the set of sequences that *f*_new _is not a part of. ({*S*_*f*_} is the union of the sequence sets of the two fragments that were aligned, which were necessarily disjoint.) For its most stringent left limit on sequence *S' *∈ {*S'*}, fragment *f*_new _considers the left limits on its immediate left neighbour on each sequence *S *∈ {*S*_*f*_} that it is a part of, and takes the most stringent of these. Say this limiting fragment on sequence *S' *is $f_{S^{\prime }}^{l}$. For this fragment, the newly-aligned fragment *f*_new _is the most stringent *right *limit for each sequence *S *∈ {*S*_*f*_}, and this applies also to every fragment to the left of $f_{S^{\prime }}^{l}$ (unless a more stringent limit already exists). This is illustrated in figure 2. So the new limits on the left can be updated in linear time; likewise for the new limits on the right.

When no further local alignments are possible, the result is a set of aligned fragments that can be "assembled" in linear time into a gapped alignment of the original sequences.

The apparent drawback here is that if, say, sequences *S*_1_, *S*_2 _and *S*_3 _are being aligned, there may be a long match between *S*_2 _and *S*_3_; but *S*_2 _may be fragmented due to earlier alignments with *S*_1_. So it may be that the match between *S*_2 _and *S*_3 _will take two or three steps (one fragment at a time) rather than one step. But this is often a gain: because we are now comparing fragments and not entire sequences, the length-dependent significance condition derived in the next section may be much improved by the reduced lengths involved. Thus, the fragmented matches will likely stay as significant as, or even be more significant than, the unfragmented match. Indeed, a match between *S*_2 _and *S*_3 _which would not be significant with an unfragmented sequence *S*_2 _may now become significant. We show in the section on synthetic data that when a random background model is assumed, Sigma does, in fact, typically align a much greater quantity of sequence than DiAlign for similar significance cutoffs, without suffering in terms of erroneous alignments.

### Scoring a local alignment

Let a local alignment of two strings *S*_1 _and *S*_2 _(respectively of lengths *L*_1 _and *L*_2_) have a length ℓ, and let there be *m *mismatches in this substring. The score we use is simply the probability of seeing such an alignment in two random sequences of the given lengths (smaller is better). DiAlign 2 [6] does this too, but somewhat differently.

Let's say *p *is the probability of two arbitrary strings of length ℓ (drawn from the same background model) matching to the same degree as the alignment we're considering: that is, having the same number of matching bases, with the bases having similar background probabilities. The value of *p *is derived below. What we need is the probability of such an alignment appearing anywhere in two sequences of lengths *L*_1 _and *L*_2_. The local alignment is made only if this probability is smaller than a predefined threshold *x *(by default 0.02).

The probability of such an alignment *not *occurring is 1 - *p *for each possible pair of subsequences of length ℓ. There are *L*_1 _- ℓ + 1 such subsequences in sequence *S*_1 _and *L*_2 _- ℓ + 1 in *S*_2_. Thus, the probability of such a match occurring *nowhere *is

$P(\text{no random matches})={\left(1-p\right)}^{\left(L_{1}-\ell +1)(L_{2}-\ell +1\right)}\;\left(1\right)$

(this is of course not exact since these probabilities are not really independent, but it is a good assumption). The probability of *at least one *random match of this quality occurring is

$\begin{array}{c} P(\text{random match})=1-{\left(1-p\right)}^{\left(L_{1}-\ell +1)(L_{2}-\ell +1\right)}\\ \approx p(L_{1}-\ell +1)(L_{2}-\ell +1)\;\left(2\right) \end{array}$

assuming *p *is small. If *p *is large, the above will be an overestimate and the match will be rejected if the cutoff threshold *x *is small, as it is by default.

DiAlign 2 [6] uses a weight function (-log *p *- *K*) which is the negative log of the above expression, but says that *"K *is a constant that depends on the sequence length" whereas for us it depends separately on *both *lengths *L*_1 _and *L*_2 _and also on the length ℓ of the local alignment. Also, *L*_1_and *L*_2 _for us are not the lengths of the original sequences but of the fragments presently being aligned, which may be much shorter, thus greatly improving the significance of a match, as noted in the previous section.

To calculate *p*, we use the product, over each matching base, of the background probability of that base, which is preferably a conditional probability reflecting *dinucleotide *counts in actual sequence. This is important since dinucleotide correlations are known to be significant in DNA, and vary from one species to another. We denote this by *p*_*bg*_. For example, for a string *"ACgCAcA" *where the base preceding this string was *T*, we use

*p*_*bg*_(*ACgCAcA*) = *p*(*A*|*T*)*p*(*C*|*A*)*p*(*C*|*G*)*p*(*A*|*C*)*p*(*A*|*C*)

skipping the factors for the lowercase letters; this is the background probability of this base pattern, with allowed mismatches at the same positions, occurring in actual sequence. (Three clarifications are needed here. First, for a base whose predecessor is a mismatched base, the geometric mean of the two conditional probabilities corresponding to the mismatches is used. Second, strictly speaking one should perhaps not use conditional probabilities for such bases at all; it is done only for efficiency reasons (the values are pre-computed and stored). Third, already-aligned sequence fragments may contain "internal" mismatches; presently, any subsequent base aligned with these is automatically treated as a mismatch. There is scope for improvement here.)

Ideally, the dinucleotide counts are taken from an auxiliary file given on the command-line. If none is given, dinucleotide counts are extracted from the input file itself, or optionally not used.

Now, the *positions *of the *m *mismatches is really arbitrary: we should multiply the above probability by the number of ways *m *mismatches can be chosen from ℓ bases. Thus, we have an additional factor of the binomial coefficient $\left(\begin{array}{l} \ell \\ m \end{array}\right)$ and our final significance expression is

$P(\text{random match})=\left(\begin{array}{l} \ell \\ m \end{array}\right)p_{\text{bg}}(L_{1}-\ell +1)(L_{2}-\ell +1).\;\left(3\right)$

If this is exceeds the threshold *x*, the alignment is rejected. (Though one may imagine that *x *= 1 should cause all local alignments to be accepted, this does not happen because the approximation in equation (2) fails. Such high values of *x *are not recommended.) DiAlign's significance parameter, given by the -thr commandline option, is analogous to - log*x*.

Early versions of Sigma experimented with "mismatch penalties" for mismatched bases (in the spirit of substitution matrices in Needleman-Wunsch-type algorithms): otherwise two random sequences can have arbitrarily long matches measured by matching bases alone. But the "entropy correction" for mismatches given by the binomial coefficient solves this problem more cleanly.

Unlike DiAlign, Sigma does not give additional weight to alignments that extend across multiple (more than two) sequences. This would be easy to implement but seems unnecessary.

## Results and discussion

### Speed

The time-complexity of the algorithm is hard to estimate exactly, but the major limiting factor is the pairwise local alignment, which is a Smith-Waterman-like dynamic-programming algorithm of O(*L*_1_*L*_2_) where *L*_1_, *L*_2 _are the lengths of the two sequence fragments being aligned. Each pass, moreover, aligns every pair of available sequence fragments. The first pass thus takes O(*L*^2^*N*^2^) time for *N *sequences of length *L*. Subsequent passes, however, align a progressively larger number of sequence fragments that grow shorter in length (decreasing *L*, increasing N); the details are quite dependent on the level of conservation in the sequences being aligned, and the total number of passes would depend on *L *and *N *in some way, but O(*L*^*p*^*N*^*q*^) with *p *and *q *not much larger than 2, seems to describe the overall time complexity in general for *L *and *N *not too large. The data structure is such that the consistency conditions can be updated at each iteration in linear time, O(*LN*). The "gaps" can be "inserted" into the sequence, prior to output, in linear time too.

An improvement in the pairwise local alignment algorithm may be possible. Related fast algorithms exist: for example, exact substring matches may be found using suffix trees, which can be constructed in linear time [17]. Suffix trees have also been used successfully for biological motif-extraction tasks (see for example [18]). Such an algorithm, if it is possible, would give a major speed boost. Another future option is parallelising the algorithm for use on clusters (which has already been done with DiAlign, as reported in [19]). In particular, the search for the best pairwise alignment is done separately over every eligible pair of fragments and would be particularly easy to parallelise.

The actual running time of Sigma on various datasets, compared to DiAlign and other programs, is discussed further below: Sigma's running time is typically close to DiAlign's, with neither program consistently faster than the other. With much larger datasets (greater than a few tens of kilobases), Sigma's speed appears to drop noticeably faster than O(*L*^2^*N*^2^) this is probably due to excessive internal fragmentation of the sequence, an issue that will be addressed in the future.

### Sensitivity and accuracy: synthetic data

A good multiple alignment program should maximise the number of bases correctly aligned (high "sensitivity"), but also minimise the number of "false positives" (high "specificity"). It may be reasonably argued that sensitivity – that is, predicting the maximum number of actually conserved structural motifs – is the major consideration in protein-alignment programs and specificity is less important. This is emphatically not true for a program aligning non-coding DNA: for example, running a motif-finder on a poor alignment could be disastrous.

To have an objective measure of the performance of various programs on data with a known evolutionary history, we focus on synthetic data in this section, but with actual genomic dinucleotide correlations. The next section discusses yeast genomic data, where direct measurement of "correctness" of the alignment is not possible.

Together with Sigma, we examine five other programs: ClustalW version 1.83 [3], MLagan version 1.21 [4], T-Coffee version 1.37 [7], Align-m version 2.3 [8], and DiAlign version 2.2.1 [6]. (Note: DiAlign has an occasional bug in its output when four or more sequences are being aligned, where two unrelated local alignments are sometimes erroneously placed on top of each other. The internal representation of the "diagonals" is not buggy, so we use a perl script, written by Michael Mwangi, to correctly assemble the fragments. Without this script, results with DiAlign are a little worse than indicated here.)

Both Sigma and DiAlign have just one adjustable parameter, the "significance threshold" beyond which local alignments are rejected. (DiAlign does use substitution matrices for proteins, while Sigma uses a correlated background model for noncoding DNA; but these are not so much "adjustable" as read or inferred from existing data.) We choose the least stringent threshold that does not erroneously align random sequence: this turns out to be about "-x 0.002" for Sigma (which we have made the default) and "-thr 6.3" for DiAlign (the default is 0.0). The number we choose for DiAlign is roughly the negative log of the number for Sigma, which is appropriate: see the section on scoring above.

In addition, Sigma was run both with a correlated background model and with a random background model.

All other programs were run with default parameters, specifying that the input is DNA sequence. MLagan requires an input binary tree specifying in what order species are to be paired; this was chosen arbitrarily, since the input data had a "flat" phylogeny (all species equidistant from ancestor) which is not supported by MLagan.

#### Generation of synthetic data

The synthetic data were generated as follows: first, an ancestral sequence was created with dinucleotide correlations drawn from real DNA sequence, possibly also containing a few embedded "motifs" drawn from "position weight matrices" meant to indicate binding sites for transcription factors, that tend to be conserved under evolution. In other words, nucleotides were laid down with conditional probabilities on the previous nucleotides, and weight matrices were laid down with a small probability; the process can be described by a hidden Markov model. This ancestral sequence was then evolved into *N *descendants, and each nucleotide was given a conservation rate *q *(that is, mutated with a probability 1 - *q*). Note that these conservation rates are from the ancestor to the descendant; the conservation rates between two descendants would be lower (*q*^2 ^rather than *q*). For example, with our chosen *q *values of 0.35, 0.45, 0.55 and 0.65, the conservation between two descendants would be 0.1225, 0.2025, 0.3025 and 0.4225 respectively.

The binding sites were assumed to be conserved (that is, the descendants were assumed to be under selection pressure at these sites); thus, if a base inside a "motif" mutated, the new base was drawn from the weight matrix representing that motif (assumed unchanged, since proteins evolve much more slowly than non-coding DNA), while if a background base mutated, the new base was drawn from the background model with a conditional (dinucleotide) probability based on the preceding base. These are precisely the assumptions made by PhyloGibbs for real data, and are justified in greater detail in [11,12]. The sequences fed to the alignment program were the *N *descendants, *not *the ancestor.

Three possibilities were considered for the background model: a completely random background (each base having probability 0.25), background dinucleotide frequencies drawn from non-coding DNA in yeast (*S. cerevisiae*), and background dinucleotide frequencies drawn from the complete genome of the malaria parasite *Plasmodium falciparum*. These genomic data are publicly available. The Plasmodium genome was picked as an extreme test case: it has an extraordinary bias to the A and T nucleotides (by far the most of any sequenced organism to date [20]) and strong codon biases even in protein coding regions. It thus provides a challenge to any multiple-alignment program.

Two further possibilities were used: plain featureless background sequence, and background sequence containing five different embedded motifs, roughly equally spaced, each of length 10 with a weight matrix "polarization" (largest element in each column) of 0.8. The motifs would act as "anchors" of strongly conserved sequence that, as we see below, help programs align the remaining sequence better.

Sequences of length 1000 bases were generated. (Typical promoters, regulatory modules or enhancer elements are a few hundred to a couple of thousand bases long, so this length is typical.) The number of sequences aligned, *N*, was taken to be 3,6,9. For each choice of all these parameters, ten runs were averaged.

## Results

The results on the "typical" case of yeast-like correlations are laid out in detail in Tables 1 and 2. With the particular parameter choices we have made, which require Sigma and DiAlign not to align random sequence, both these programs align very little sequence erroneously, for either weakly-conserved or relatively strongly-conserved sequence. In contrast, it is immediately apparent that, with weakly conserved sequence *(q = *0.35 or 0.45), ClustalW, MLagan and T-Coffee – which are all based on Needleman-Wunsch-type approaches, with gap penalties and gap-extension penalties – align far too much sequence erroneously. Changing the gap penalties either had no significant effect, or had other problems such as excessively-fragmented alignments. Perhaps a non-default substitution matrix would improve things, but in our opinion, Needleman-Wunsch-type approaches are not well suited for the problem of non-coding DNA, where large regions may be well conserved but equally large regions may show no conservation at all.

**Table 1:** Results on synthetic yeast-like data. Performance of various multiple sequence alignment programs on synthetic data generated with dinucleotide correlations that mimic actual yeast genomic data. *q *is the "proximity" of the species to their common ancestor, ie the probability that a given base is conserved from its common ancestor. This means that *q*^2 ^is the conservation rate of bases in any pair of descendants. *N*_+ _is the number of bases correctly aligned. *N*_- _is the number of bases incorrectly aligned. Each data set consisted of 10 sets each containing *N*_*s *_sequences, each 1000 bases long, so the number of bases is 10000*N*_*S*_. Sen is the sensitivity, ie the ratio of number of bases correctly aligned to total number of bases, *N*_+_/(10000*N*_*s*_). Er is the error rate, *N*_-_/(*N*_+ _+ N_-_). sigma+ indicates sigma with a background model incorporating dinucleotide correlations. sigma— indicates sigma with an uncorrelated background model.

		*q *= 0.35				*q *= 0.45				*q *= 0.55				*q *= 0.65			
No embedded WM's																	

*N*_*s*_	Prog	*N*_+_	*N*_-_	Sen	Er	*N*_+_	*N*_-_	Sen	Er	*N*_+_	*N*_-_	Sen	Er	*N*_+_	*N*_-_	Sen	Er

3	sigma+	0	0	0.00	N/A	166	0	0.01	0.00	3210	0	0.11	0.00	28755	0	0.96	0.00
	sigma-	0	0	0.00	N/A	761	0	0.03	0.00	10737	0	0.36	0.00	29606	0	0.99	0.00
	dialign	0	0	0.00	N/A	266	0	0.03	0.00	854	0	0.04	0.00	9115	0	0.30	0.00
	alignm	320	136	0.01	0.30	6009	846	0.20	0.12	22710	1083	0.76	0.05	28258	434	0.94	0.02
	clustalw	15244	14756	0.51	0.49	25659	4341	0.86	0.14	28959	1041	0.97	0.03	29779	221	0.99	0.01
	mlagan	13691	16309	0.46	0.54	24766	5234	0.83	0.17	29596	404	0.99	0.01	30000	0	1.00	0.00
	tcoffee	3781	26219	0.13	0.87	15253	14747	0.51	0.49	26542	3458	0.88	0.12	29759	241	0.99	0.01
6	sigma+	74	0	0.00	0.00	334	0	0.01	0.00	27765	24	0.46	0.00	57820	0	0.96	0.00
	sigma -	74	112	0.00	0.60	590	0	0.01	0.00	42882	40	0.71	0.00	58948	0	0.98	0.00
	dialign	66	158	0.00	0.71	604	176	0.01	0.23	7364	114	0.12	0.02	30871	0	0.51	0.00
	alignm	0	0	0.00	N/A	7192	123	0.12	0.02	53534	978	0.89	0.02	59326	222	0.99	0.00
	clustalw	29878	30122	0.50	0.50	52295	7705	0.87	0.13	57712	2288	0.96	0.04	59580	420	0.99	0.01
	mlagan	17411	42589	0.29	0.71	48105	11895	0.80	0.20	58736	1264	0.98	0.02	60000	0	1.00	0.00
	tcoffee	13215	46785	0.22	0.78	41965	18035	0.70	0.30	58084	1916	0.97	0.03	59925	75	1.00	0.00
9	sigma+	0	0	0.00	N/A	597	0	0.01	0.00	41873	40	0.47	0.00	87769	0	0.98	0.00
	sigma -	160	64	0.00	0.29	2577	162	0.03	0.06	63579	228	0.71	0.00	88764	0	0.99	0.00
	dialign	78	264	0.00	0.77	1045	228	0.01	0.18	12162	176	0.14	0.01	54753	0	0.61	0.00
	alignm	44	159	0.00	0.78	29033	460	0.32	0.02	83545	960	0.93	0.01	89261	330	0.99	0.00
	clustalw	52761	37239	0.59	0.41	79733	10267	0.89	0.11	86758	3242	0.96	0.04	89429	571	0.99	0.01
	mlagan	16445	73555	0.18	0.82	68421	21579	0.76	0.24	88828	1172	0.99	0.01	90000	0	1.00	0.00
	tcoffee	27005	62995	0.30	0.70	67009	22991	0.74	0.26	88534	1466	0.98	0.02	89955	45	1.00	0.00

**Table 2:** More results on synthetic yeast-like data. Same as Table 1, except that the sequences have five embedded motifs (drawn from weight matrices that had 80% "polarisation"); this better mimics real data and also improves the performance of all programs.

		*q *= 0.35				*q *= 0.45				*q *= 0.55				*q *= 0.65			
5 embedded WM's																	

*N*_*s*_	Prog	*N*_+_	*N*_-_	Sen	Er	*N*_+_	*N*_-_	Sen	Er	*N*_+_	*N*_-_	Sen	Er	*N*_+_	*N*_-_	Sen	Er

3	sigma+	0	0	0.00	N/A	0	0	0.00	N/A	13266	0	0.44	0.00	29352	0	0.98	0.00
	sigma-	0	0	0.00	N/A	456	0	0.02	0.00	19028	0	0.63	0.00	29274	0	0.98	0.00
	dialign	78	0	0.03	0.00	60	0	0.02	0.00	2639	0	0.09	0.00	11383	40	0.38	0.00
	alignm	3067	506	0.10	0.14	11389	2153	0.38	0.16	24496	1217	0.82	0.05	28687	305	0.96	0.01
	clustalw	20814	9186	0.69	0.31	26730	3270	0.89	0.11	29341	659	0.98	0.02	29878	122	1.00	0.00
	mlagan	17160	12840	0.57	0.43	26930	3070	0.90	0.10	29716	284	0.99	0.01	30000	0	1.00	0.00
	tcoffee	10421	19579	0.35	0.65	20548	9452	0.68	0.32	28129	1871	0.94	0.06	29887	113	1.00	0.00
6	sigma+	0	0	0.00	N/A	1625	0	0.03	0.00	36055	28	0.60	0.00	58596	0	0.98	0.00
	sigma-	452	42	0.01	0.09	5984	0	0.10	0.00	47264	26	0.79	0.00	58876	0	0.98	0.00
	dialign	510	144	0.01	0.22	1719	74	0.03	0.04	10250	0	0.17	0.00	33979	0	0.57	0.00
	alignm	3893	181	0.06	0.04	28300	1363	0.47	0.05	54487	1077	0.91	0.02	59496	170	0.99	0.00
	clustalw	42116	17884	0.70	0.30	53797	6203	0.90	0.10	58316	1684	0.97	0.03	59613	387	0.99	0.01
	mlagan	29458	30542	0.49	0.51	53833	6167	0.90	0.10	59427	573	0.99	0.01	60000	0	1.00	0.00
	tcoffee	25878	34122	0.43	0.57	47767	12233	0.80	0.20	58708	1292	0.98	0.02	59975	25	1.00	0.00
9	sigma+	40	30	0.00	0.43	2300	0	0.03	0.00	56847	20	0.63	0.00	87436	0	0.97	0.00
	sigma-	322	30	0.00	0.09	5805	0	0.06	0.00	69821	120	0.78	0.00	88775	0	0.99	0.00
	dialign	450	80	0.01	0.15	2844	106	0.03	0.04	20179	0	0.22	0.00	59400	0	0.66	0.00
	alignm	10165	652	0.11	0.06	60374	2420	0.67	0.04	85011	933	0.94	0.01	89651	201	1.00	0.00
	clustalw	62461	27539	0.69	0.31	83233	6767	0.92	0.08	86738	3262	0.96	0.04	89167	833	0.99	0.01
	mlagan	33857	56143	0.38	0.62	81225	8775	0.90	0.10	88869	1131	0.99	0.01	90000	0	1.00	0.00
	tcoffee	43416	46584	0.48	0.52	76928	13072	0.85	0.15	89240	760	0.99	0.01	89990	10	1.00	0.00

Align-m, on the other hand, aligns remarkably little sequence erroneously; for weakly conserved sequence it shows improved sensitivity over Sigma and DiAlign (but has a somewhat higher error rate than Sigma), and does vastly better than ClustalW, MLagan and T-Coffee in terms of error rate. Its main drawbacks are its running time and its memory consumption, which are an order of magnitude more than other programs and rise sharply with the number of sequences being aligned.

At *q *= 0.55 Sigma and DiAlign continue to have a lower sensitivity than the other programs, but Sigma also has a much lower error rate. At *q *= 0.65, all programs show high sensitivity (DiAlign's is noticeably lower than the rest) and low error rates (Sigma makes no errors at all).

This is seen even more sharply with the highly-correlated plasmodium-like numbers, presented in brief in table 3. With completely uncorrelated sequence where each nucleotide has a probability 0.25, the gaps between the various algorithms narrow but the trends remain visible (table 4).

**Table 3:** Results on synthetic plasmodium-like data. Performance of various multiple sequence alignment programs on synthetic data generated with dinucleotide correlations that mimic the *Plasmodium falciparum *genome. See the caption of Table 1 for explanation of the column and row labels.

		*q *= 0.35				*q *= 0.45				*q *= 0.55				*q *= 0.65			
No embedded WM's																	

*N*_*s*_	Prog	*N*_+_	*N*_-_	Sen	Er	*N*_+_	*N*_-_	Sen	Er	*N*_+_	*N*_-_	Sen	Er	*N*_+_	*N*_-_	Sen	Er

3	sigma+	0	0	0.00	N/A	0	0	0.00	N/A	1036	0	0.03	0.00	24617	0	0.82	0.00
	sigma-	66	1070	0.00	0.94	9175	372	0.31	0.04	27959	0	0.93	0.00	29547	0	0.98	0.00
	dialign	292	1471	0.01	0.83	800	1582	0.03	0.66	4185	929	0.14	0.18	14957	104	0.50	0.01
	alignm	753	1563	0.03	0.67	5889	2085	0.20	0.26	20462	1846	0.68	0.08	27433	728	0.91	0.03
	clustalw	12057	17943	0.40	0.60	23528	6472	0.78	0.22	27965	2035	0.93	0.07	29814	186	0.99	0.01
	mlagan	7974	22026	0.27	0.73	21709	8291	0.72	0.28	29145	855	0.97	0.03	30000	0	1.00	0.00
	tcoffee	6865	23135	0.23	0.77	15398	14602	0.51	0.49	25261	4739	0.84	0.16	29043	957	0.97	0.03
6	sigma+	0	0	0.00	N/A	48	0	0.00	0.00	8033	0	0.13	0.00	54293	0	0.90	0.00
	sigma-	1684	8161	0.03	0.83	26675	4113	0.44	0.13	57619	86	0.96	0.00	59411	0	0.99	0.00
	dialign	294	7229	0.00	0.96	3423	5362	0.06	0.61	15806	2521	0.26	0.14	46203	75	0.77	0.00
	alignm	0	50	0.00	1.00	3678	237	0.06	0.06	42406	972	0.71	0.02	57938	610	0.97	0.01
	clustalw	26172	33828	0.44	0.56	46240	13760	0.77	0.23	55270	4730	0.92	0.08	59234	766	0.99	0.01
	mlagan	13737	46263	0.23	0.77	40430	19570	0.67	0.33	57668	2332	0.96	0.04	59931	69	1.00	0.00
	tcoffee	19149	40851	0.32	0.68	40764	19236	0.68	0.32	56346	3654	0.94	0.06	59532	468	0.99	0.01

**Table 4:** Results on uncorrelated data. Performance of various multiple sequence alignment programs on synthetic data with no dinucleotide correlations, and each base having probability 0.25. See the caption of Table 1 for explanation of the column and row labels.

		*q *= 0.35				*q *= 0.45				*q *= 0.55				*q *= 0.65			
No embedded WM's																	

*N*_*s*_	Prog	*N*_+_	*N*_-_	Sen	Er	*N*_+_	*N*_-_	Sen	Er	*N*_+_	*N*_-_	Sen	Er	*N*_+_	*N*_-_	Sen	Er

3	sigma-	0	0	0.00	N/A	260	0	0.01	0.00	11690	0	0.39	0.00	29154	0	0.97	0.00
	dialign	0	60	0.00	1.00	80	0	0.03	0.00	1000	0	0.06	0.00	7566	0	0.25	0.00
	alignm	0	0	0.00	N/A	6682	605	0.22	0.08	23338	656	0.78	0.03	28466	283	0.95	0.01
	clustalw	16675	13325	0.56	0.44	26450	3550	0.88	0.12	29340	660	0.98	0.02	29828	172	0.99	0.01
	mlagan	11280	18720	0.38	0.62	26874	3126	0.90	0.10	29767	233	0.99	0.01	30000	0	1.00	0.00
	tcoffee	3443	26557	0.11	0.89	12475	17525	0.42	0.58	27836	2164	0.93	0.07	29810	190	0.99	0.01
6	sigma-	0	0	0.00	N/A	0	0	0.00	N/A	23583	0	0.39	0.00	58986	0	0.98	0.00
	dialign	0	0	0.00	N/A	138	0	0.01	0.00	4588	0	0.08	0.00	28177	0	0.47	0.00
	alignm	0	0	0.00	N/A	13090	174	0.22	0.01	53252	824	0.89	0.02	59529	139	0.99	0.00
	clustalw	37040	22960	0.62	0.38	53438	6562	0.89	0.11	58432	1568	0.97	0.03	59727	273	1.00	0.00
	mlagan	15987	44013	0.27	0.73	50702	9298	0.85	0.15	58621	1379	0.98	0.02	59917	83	1.00	0.00
	tcoffee	13439	46561	0.22	0.78	35387	24613	0.59	0.41	58292	1708	0.97	0.03	59941	59	1.00	0.00

Embedding a few highly-conserved weight matrices (as in table 2) improves the performance of all the programs, compared to table 1, but does not greatly change their relative performances.

In addition to the Sigma and DiAlign results shown, we tried the parameters "-x 0.02" and "-x 0.2" for Sigma, "-thr 3.9" and "-thr 1.6" for DiAlign. While a less stringent threshold improves sensitivity only slightly, it decreases specificity significantly (particularly with weakly-conserved sequence), which is an important criterion for us.

In summary, on synthetic data, with strong conservation (*q *≈ 0.65 or more) the difference between various algorithms is marginal, but with weak or intermediate conservation Sigma, DiAlign and Align-m all make significantly fewer erroneous alignments than other algorithms; and of these, Sigma generally shows better sensitivity than DiAlign, and a lower error rate than either DiAlign or Align-m (it is also far faster and less memory-intensive than Align-m).

The poor sensitivity of Sigma on weakly-conserved sequence (and the high error rates of programs that do align significant amounts of such sequence) suggests that it is a very hard problem to accurately align uniformly-diverged non-coding DNA. In real life, however, regions of well-conserved DNA may be interspersed with regions of poorly-conserved DNA. Here, we would be helped by Sigma's approach of using only the length of the fragments being aligned to judge the significance of an alignment. Table 2, for *q *= 0.45, shows a nearly 5-fold better performance of sigma+ (ie, with correlations accounted for) when some highly-conserved "motifs" are embedded in a poorly-conserved background. Other programs, too, improve in performance, but not as sharply.

Benchmarks of any kind must always be viewed with some skepticism. The most realistic head-to-head comparison here is with DiAlign, and this comparison most clearly shows the improvements from Sigma's algorithm and scoring in specificity and error rate. It is possible that, with fine-tuning of parameters, other programs may perform much better than shown here.

### Yeast genomic data

It is of great interest to measure the performance of different alignment programs on real genomic data, but the problem is that we don't know the "correct" answer. In protein sequence, structural information may give clues to what sequence is likely to have been phylogenetically conserved. Non-coding sequence evolves much faster, and binding sites are known not always to be conserved-instead, they tend to come and go, at least in yeast and drosophila [21,22]. However, we get some suggestions by examining the performance of our recently developed motif finder, PhyloGibbs [12] on these alignments. Since a primary goal of Sigma is to aid motif-finding and similar tasks, this is a relevant, if indirect, benchmark.

We use a database, the Saccharomyces cerevisiae promoter database (SCPD) [23], that documents experimentally verified binding sites for yeast transcription factors. Since this database is not computationally derived, it serves as an unbiased real-world benchmark, and we also used it to measure the performance of PhyloGibbs itself [12]. After some elimination of excessively long or redundant sites, we were left with 466 annotated binding sites upstream of 200 different genes.

Regulatory regions for these genes from *S. cerevisiae *and, where available, orthologues from (in order of closeness to *S. cerevisiae*) *S. paradoxus *[10], *S. mikatae, S. kudriavzveii and S. bayanus *[9] were used. All data is publicly available from SGD [24]. The binary tree input for MLagan used the same order of closeness above. Only Sigma alignments incorporating yeast-like correlated background model were used. Other than this, all alignments were generated with the same parameters as in the previous section. For the purposes of PhyloGibbs, it was assumed that the yeast species are equidistant from their last common ancestor, with a "proximity" (probability that a base not under selection is unchanged from the ancestor) of 0.5. The complete PhyloGibbs commandline was: -D 1 -G 0.5 -f *inputfile *-o *outputfile *-t *trackingfile *-m 10 -N 1 -F *backgroundfile *-I 3,3,3, which means that it assumes phylogenetic alignment of the input sequences with uniform proximity 0.5, a dinucleotide background model extracted from the file *backgroundfile*, and an initial guess of three different transcription factors each having three (possibly multi-species) binding sites, each site 10 bases long. These are the same parameters used and explained further in [12].

PhyloGibbs uses a two-stage process of motif-finding, a simulated anneal to find the "best answer" followed by Markov-chain Monte-Carlo sampling of the entire configuration space and statistical "tracking" to find the "significance" of the answer (and possibly, also, of other sites not reported in the simulated anneal). It thus assigns to each reported binding site a "tracking score" *t *(between 0 and 1) indicating its probability of being a genuine motif. As a function of *t*, there are two quantities of interest to us: the number of predictions of PhyloGibbs that are documented binding sites in *S. cerevisiae *(predictions in other species are ignored for this purpose), and the ratio of these to the total number of predictions. These may be taken as a measure of the sensitivity and specificity, respectively, of PhyloGibbs. Since the SCPD data is far from exhaustive, these numbers must be taken only as indicative trends: the "false positive" rate cannot really be measured. Further discussion is in the PhyloGibbs paper [12].

Of these, the specificity is more relevant: by increasing the total number of predictions, the sensitivity can always be increased, to a point where predictions are meaningless. Moreover, the sensitivity here is of PhyloGibbs's predictions and not of the input alignments; this is hard to interpret, whereas one may reasonably argue that high specificity in PhyloGibbs predictions indicates more generally correct alignments.

**Figure 3:**
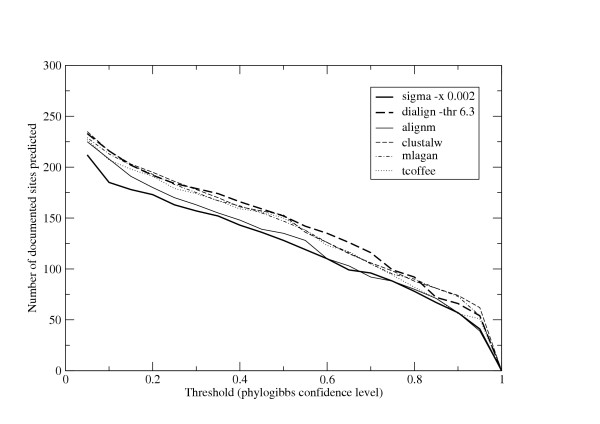
**Sensitivity of PhyloGibbs with different alignments**. The total number of documented binding sites predicted by Phylogibbs run on various alignments: a measure of the sensitivity of PhyloGibbs on those alignments.

**Figure 4:**
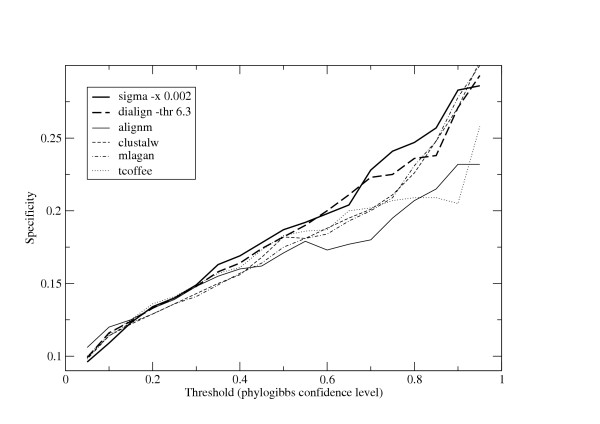
**Specificity of PhyloGibbs with different alignments**. The ratio of predictions that are documented binding sites, to total predictions, by Phylogibbs run on various alignments: a measure of the specificity of PhyloGibbs on those alignments, and indirectly a measure of the quality of those alignments.

The results are plotted in figures 3 and 4. As in the synthetic data, the best results are obtained with the options -x 0.002 for Sigma and -thr 6.3 for DiAlign. For most threshold cutoffs, PhyloGibbs on Sigma alignments is inferior on sensitivity but clearly outperforms all the other programs on specificity: in other words, with Sigma there are fewer predictions overall but these are made with higher confidence. This is especially true at the high-threshold end, where sensitivities of all programs are somewhat comparable but the specificity of Sigma runs is considerably higher. Surprisingly, AlignM, which was generally a good performer on the synthetic data, performs comparatively poorly here on both sensitivity and specificity. As in the previous section, this data should be taken as suggestive but not conclusive. Programs other than Sigma and DiAlign are run with default parameters that are certainly unsuitable for non-coding DNA. Moreover, the total fraction of sequence aligned by these programs is different (as was shown in the previous section), and this too somewhat affects the predictions of PhyloGibbs, for reasons too complex to go into here.

### Running times

**Figure 5:**
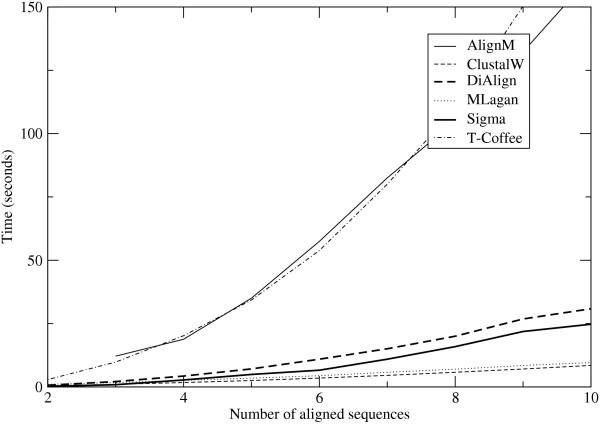
**Running time of different alignment programs**. Six alignment programs are run on the plasmodium-like synthetic sequence with *q *= 0.55 (see caption of tables 1 and 2), on *N *sequences of length l000 bp each; for *N *= 2–10. (The AlignM program requires at least 3 input sequences.)

Figure 5 shows the running times for the six programs studied here as a function of number of input sequences being simultaneously aligned, each of length 1000 bp. (The DiAlign numbers here are for the program binary which outputs erroneous alignments, as mentioned in the section on synthetic data. The correction script in perl that we use in practice increases the running time by a factor of 5 or 10.) The fastest programs are ClustalW and MLagan, followed with some gap by Sigma and DiAlign. AlignM and T-Coffee are an order of magnitude slower. (AlignM also consumes a lot of memory—well over 100 MB when aligning more than 7 sequences.) It appears that T-Coffee scales the most poorly of these six programs to large numbers of sequences.

The results on the yeast data are similar except that DiAlign is slightly faster than Sigma, while AlignM is almost twice as slow as T-Coffee. The total running times for the alignment programs in seconds, for aligning 184 gene promoters for which at least three orthologous sequences existed (a requirement for AlignM), were - AlignM: 1850.2; ClustalW: 168.21; DiAlign: 255.52; MLagan: 430.7; Sigma: 266.5; T-Coffee: 646.45.

Thus, on data sets of this size, Sigma's speed is competitive with three of the other five programs studied, and significantly better than the remaining two, without compromising on accuracy and sensitivity.

## Conclusion

The present implementation of Sigma focuses on programming simplicity rather than speed—the complete source code, excluding comments, totals under 1000 lines in Objective Caml (a functional language of the ML family, available from http://caml.inria.fr). Nevertheless, it is already a fully-functional tool competitive with or superior to other programs in speed and accuracy, and substantial improvements in both respects may be possible. However, the present version is intended to be a starting point for more challenging tasks, some of which are in progress.

Pairwise alignment programs, like other tools in bioinformatics, are not error-free and Sigma is no exception. As remarked above, the output of such programs is used as the input for other tasks such as regulatory site prediction; it would be interesting to build an integrated tool that not only predicts regulatory sites (or modules or enhancers) based on a multiple alignment, but uses the information thus obtained to in turn improve the multiple alignment. Such an approach may have far more success both in aligning weakly-conserved sequence and in predicting functional sites in that sequence, but would also be far more complex than either a stand-alone sequence-alignment program or a stand-alone motif-finder.

A general assumption in multiple-alignment algorithms is that synteny is preserved among the aligned pieces. This assumption, quite reasonable for most proteins, grows progressively more dubious for longer stretches of non-coding DNA and it may be desirable to relax it in a controlled manner (the problem would be the hugely increased "search space" of possible matches).

Thus, the hope is that Sigma can be significantly improved and extended in the future, and interfaced with other tools such as motif-finders and module-prediction programs.

## Availability and requirements

**Project name: **Sigma

**Project home page: **http://www.imsc.res.in/~rsidd/sigma/

**Operating systems: **Binaries for Linux and Windows available; source may be compiled on any system supported by Objective Caml

**Programming language: **Objective Caml (version 3.x)

**Other requirements: **None

**Licence: **GNU GPL

**Any restrictions to use by non-academics: **None

## Competing interests

The author(s) declares that they have no competing interests.
